# Multi‐Functional Smart Skin for High‐Efficiency Energy‐Saving Buildings

**DOI:** 10.1002/advs.78166

**Published:** 2026-10-06

**Authors:** Zongxu Na, Li Yu, Bo Lu, Jing Li, Xianyang Yue, Qian Wang

**Affiliations:** ^1^ State Key Laboratory of Green and Efficient Development of Phosphorus Resources Hubei Key Laboratory of Plasma Chemistry and Advanced Materials School of Materials Science and Engineering Wuhan Institute of Technology Wuhan People's Republic of China; ^2^ Institute for Advanced Materials and Technology University of Science and Technology Beijing Beijing People's Republic of China; ^3^ School of Printing and Packaging Engineering Beijing Institute of Graphic Communication Beijing People's Republic of China

**Keywords:** energy‐saving buildings, multi‐functional, multi‐responsive shape‐memory effect, smart skin, thermal management

## Abstract

Exploring scalable smart devices with broadband modulation, zero‐energy input (without an external energy supply) controllable operation mode, and thermal insulation is of great significance for the building energy‐saving. However, it remains a grand challenge to integrate such essential multi‐functionality into one single device for affording extraordinary energy‐saving effect. Here, we present a single‐layer structured multi‐functional smart “skin” (MSS) composed of thermal managing unit of high solar reflectance (*R*_sol_ = 93.8%) and low thermal conductivity (ĸ = 54 mW/m·K) and actuation unit of multi‐responsive shape‐memory effect. The ingenious integration of static thermal radiation property and dynamic shape changing capability enables the MSS superb solar modulating ability (Δ*T*_sol_ = 88.1%). Selecting twenty‐two cities with distinct climate conditions as the sample for analysis, the average energy‐saving efficiency and carbon emission reduction of the MSS is 26.7% and 58.3 kg/m^2^. In addition, the MSS possesses zero‐input controllable operation mode in all‐season as assembling with a perovskite solar cell. This concept promotes the application of advanced functional devices in buildings, automobiles, and smart textiles.

## Graphical abstract

A scalable multifunctional smart skin (MSS) with broadband modulation, zero‐energy input controllable operation mode, and thermal insulation is designed by assembling thermal managing unit of high solar reflectance and low thermal conductivity and actuation unit of multi‐responsive shape‐memory effect. The MSS exhibits superb energy‐saving effect in diverse climatic regions because essential multi‐functionality is integrated into one single device.

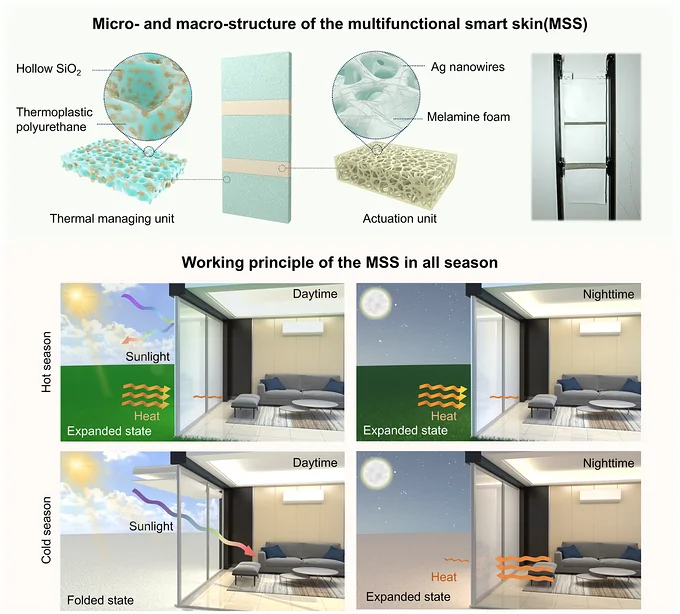

## 1 Introduction

Buildings take essential role in human daily life because they are able to provide an independent, comfortable, and safe working and living space for human beings. With continuous deterioration of environmental climate, the building energy consumption including indoor heating and air‐conditioning grows with each passing day [1]. Therefore, energy‐saving buildings (ESBs), a green technology is capable of effectually decreasing the building energy consumption by regulating and harnessing solar energy, have become a research hotspot across the fields of nanomaterial, energy, artificial intelligence [2, 3].

Windows are the main medium for the energy exchange between ESBs and external environment [4]. Taking consideration of natural lighting, structural optimization, and aesthetic, the proportion of windows displays sustained growth in modern architectures, especially in high‐rise buildings [5]. Recently, a variety of thermal‐, electrical‐, and mechanical‐responsive smart devices that are able to dynamically modulate their solar transmittance (*T*_sol_) have been designed for exploiting ESBs [6, 7]. Generally, these stimuli‐responsive devices are based on smart materials such as transition metal oxides (VO_2_, WO_3_) [8, 9, 10, 11, 12, 13], hydrogels [14, 15, 16, 17], conducting polymers [18, 19], and liquid crystals [20, 21, 22, 23, 24] and smart structures [25, 26, 27]. Particularly, thermal‐responsive smart devices possess energy‐free Δ*T*_sol_ attributed to their temperature sensing capability [28]. However, the temperature dependence characteristic makes their *T*_sol_ cannot be freely manipulated according to the demand of the occupant under different weather conditions [29]. This issue does not occur in electrical‐responsive smart devices because their *T*_sol_ is able to be accurately modulated by external voltage [30]. Commonly, electrical‐responsive smart devices are composed of transparent conducting electrode, electrochromic material, and liquid electrolyte [31]. The multi‐layer structure results in complex preparation process, relatively higher cost, and difficulty in achieving uniform performance in large‐scale [32]. Although mechanical‐responsive smart devices combine energy‐free and freely‐controlled operation mode, large strain or narrow‐band modulation behavior restricts their practical application [33, 34].

For building high‐efficiency ESBs, several critical characteristics are indispensable for smart devices. Based on the energy distribution of solar radiation [35], broadband modulation behavior and high Δ*T*_sol_ are required for achieving ideal energy‐saving effect [36, 37]. The energy exchange between ESBs and external environment through windows is quite complex, including conduction, convection, and thermal radiation [38]. Accordingly, the thermal insulating performance, playing a significant role in energy‐saving effect, also should be involved in the design of smart devices [39]. In addition, the operation mode of smart devices should be completely controllable which means the *T*_sol_ of smart devices can be freely manipulated according to the demand of the occupant under different weather conditions due to basic function of windows, connecting indoor and outdoor views [40, 41]. Last but not least, they are low cost and facile to be manufactured in large‐scale, holding a great potential for real‐world application [42, 43]. These characteristics are particularly important for smart devices to develop high‐efficiency ESBs, but never all realized in the existing smart devices.

Herein, we report a single‐layer structured MSS with broadband modulation, all‐day controllable operational mode, and low thermal conductivity, affording ESBs extraordinary energy‐saving effect in all‐season. As presented in Figure 1A, the MSS is composed of two key components: thermal managing and actuation units. The thermal managing unit is hollow SiO_2_/polyurethane (HSPU) porous nanocomposite film of high solar reflectance (*R*_sol =_ 93.8%) and low thermal conductivity (ĸ = 54 mW/m·K). The actuation unit is melamine foam/Ag/polyurethane composite film with multi‐responsive shape‐memory effect (Figure 1B) [44, 45]. The multi‐responsive shape‐memory effect can enable the MSS adjustable operational mode, expanded and folded states, under complex weather conditions, which will be detailed elucidated in Figures 3 and 4. Our design ingeniously integrates static high solar reflection of the thermal managing unit and multi‐responsive shape changing behavior of the actuation unit, offering the MSS exceptional broadband modulation capability (Figure 1C). The Δ*T*_sol_ of the MSS reaches up to 88.1%, which displays a relatively high level compared to currently reported smart devices (Figure 1D). In daytime at hot season, the MSS at expanded state is able to effectively block the thermal radiation of solar energy into the indoor attributed to high solar reflectance (*R*_sol =_ 93.8%), remarkably reducing the cooling energy consumption (Figure 1Ei). Moreover, the energy‐saving performance can be further elevated because the MSS of low thermal conductivity (ĸ = 54 mW/m·K) can efficaciously curb the heat exchange between the indoor and outdoor environment. The thermal insulation effect of the MSS works in daytime as well as nighttime (Figure 1Eii). In daytime at cold season, the thermal irradiation of solar energy can be employed to compensate the energy consumption for the indoor heating due to high *T*_sol_ = 88.2% of the MSS at folded state (Figure 1C and Figure 1Eiii). In nighttime at cold season, the heating energy consumption can be suppressed caused by the thermal insulation effect of the MSS at expanded state (Figure 1Eiv).

**FIGURE 1:**
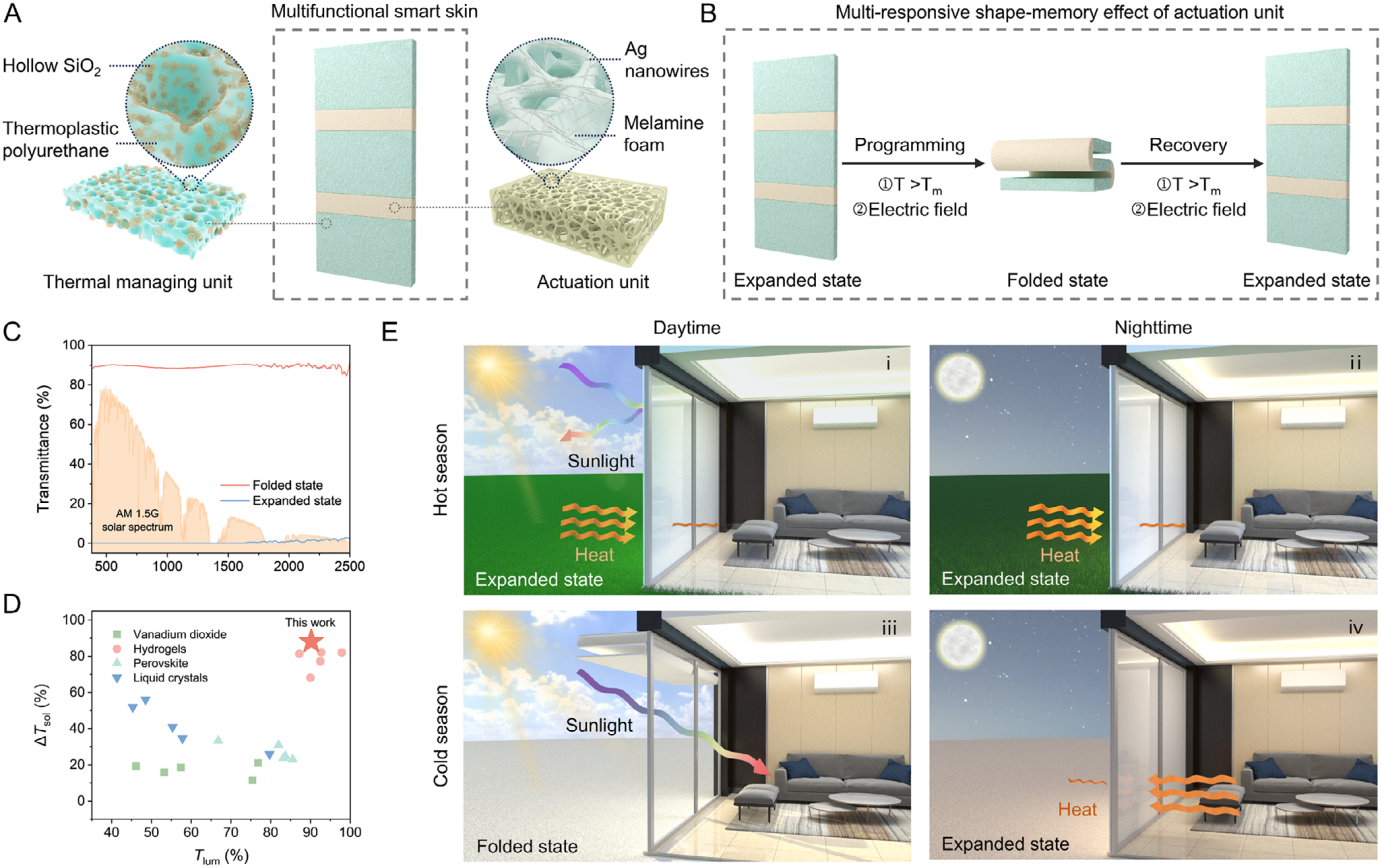
(A) Schematic of the macro‐/micro‐structure of the MSS. (B) Multi‐responsive shape‐memory effect of the actuation unit for offering the MSS adjustable operational mode under complex weather conditions. (C) Transmittance spectra of (0.38–2.5 µm) the MSS at expanded and folded states. (D) Summaries of the Δ*T*_sol_ of recently reported smart devices (Table S3). (E) Operation principle of the MSS in hot and cold seasons.

## 2 Results and Discussion

### 2.1 Fabrication and Optimization of Thermal Managing Unit

To prove our concept, porous HSPU nanocomposite film acting as the thermal managing unit is fabricated by a simple, scalable, and inexpensive nonsolvent induced phase separation method via using commercially available thermoplastic polyurethane (TPU) and hollow SiO_2_ as the polymer matrix and functional component respectively (Figure 2A,B, see Methods for details) [46]. The HSPU nanocomposite film exhibits hierarchically porous structure that is abundant in nano‐ and micro‐pores (Figure 2C), enabling the thermal managing unit high solar reflectance and low thermal conductivity [47, 48]. The incorporation of hollow SiO_2_ nanoparticles with the diameter around 305 nm is conducive to further enhancing the solar reflectance and lowering the thermal conductivity because of their unique microstructure (Figure 2D,E) [49, 50]. To optimize the performance of the thermal managing unit, the influence of the concentration of the TPU precursor solution and loading of the hollow SiO_2_ nanoparticles on the solar reflectance and thermal conductivity of the porous HSPU nanocomposite film is systematically investigated. As presented in Figure S1A–C, the porous TPU film possesses the highest solar reflectance *R*_sol_ = 91.0% and lowest thermal conductivity ĸ = 61 mW/m·K when the concentration of the TPU precursor solution is 10.0 wt.% resulted from relatively higher porosity 63.4% and uniform and dense porous microstructure (Figure S1D and Figure 2). The introduction of hollow SiO_2_ nanoparticles can improve the solar reflectance of the porous HSPU nanocomposite film owing to their scattering effect [51]. The solar reflectance reaches as high as 93.8% as the loading of the hollow SiO_2_ nanoparticles is 1.5 wt.% (Figure 2F). However, the scattering effect of the hollow SiO_2_ nanoparticles becomes weak when their loading is over 1.5 wt.% because micrometer size hollow SiO_2_ aggregates appear (Figure S3A–E) [52]. The micrometer size hollow SiO_2_ aggregates clog the pores formed by nonsolvent induced phase separation method, decreasing the porosity of the HSPU nanocomposite film (Figure S3F). This is accordance with the phenomenon that the HSPU nanocomposite film displays the best thermal insulation performance (lowest thermal conductivity ĸ = 54 mW/m·K) when the loading of the hollow SiO_2_ nanoparticles is 1.5 wt.% (Figure 2G) [53]. To directly present the thermal insulation property of the HSPU nanocomposite film, a sandwich structure sample in which the HSPU nanocomposite film is placed between two pieces of normal glass as an insulating layer is put on a heating table set at 60°C. Meanwhile, an infrared thermal camera is applied to monitor the surface temperature of the sandwich structure sample. In addition, a piece of glass (ĸ = 91 mW/m·K) with the same thickness as the HSPU nanocomposite film is chosen as the reference (Figure 2H). As presented in Figure 2I, the surface temperature of the sandwich structure sample in which the HSPU nanocomposite film (HS_1.5_PU_10_) is used as the insulating layer is 44°C at stable state. The 44°C is 16°C and 10°C lower than the temperature of the heating table and the reference sample respectively, indicating superb thermal insulation effect of the HSPU nanocomposite film (HS_1.5_PU_10_). The self‐made setup tests further prove that the HSPU nanocomposite film exhibits the best thermal insulation performance as the loading of the hollow SiO_2_ nanoparticles is 1.5 wt.% (Figure S4). Comprehensive consideration of the solar energy reflecting and thermal insulation property, the HS_1.5_PU_10_ is selected as the thermal managing unit to build up the MSS. Moreover, the thermal managing unit with a large size (20 cm × 20 cm) is fabricated (Figure S5A). The solar reflectance and thermal conductivity of three distinct areas in the thermal managing unit are tested. There is no significant difference in the solar energy reflecting and thermal insulation performance of these three distinct areas, verifying the uniform of the thermal managing unit (Figure S5B,C).

**FIGURE 2:**
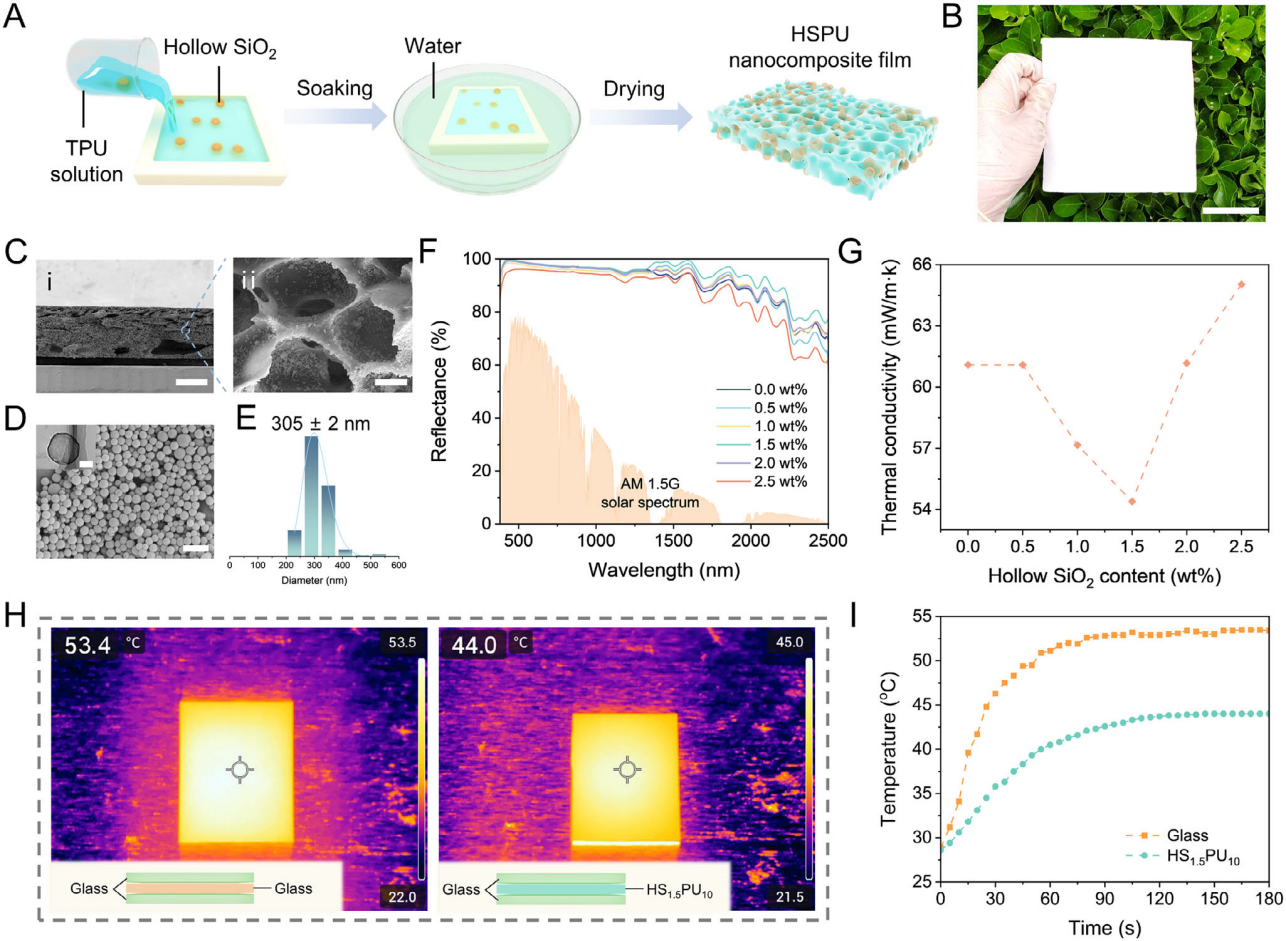
(A) Schematic of the nonsolvent induced phase separation method for fabricating the porous HSPU nanocomposite film. (B) Image displaying a laboratory‐manufactured, scalable, and flexible porous HSPU nanocomposite film. The scale bar is 5 cm. (C) Cross‐section scanning electron microscope (SEM) images of the porous HSPU nanocomposite film (HS_1.5_PU_10_). The scale bar of the (i) and (ii) is 300 µm and 5 µm, respectively. (D) SEM and transmission electron microscope (TEM) image of the hollow SiO_2_ nanoparticles. The scale bar is of SEM and TEM is 1 µm and 10 nm, respectively. (E) Size distribution of the hollow SiO_2_ nanoparticles. (F) Reflectance and (G) thermal conductivity of the porous HSPU nanocomposite films with different concentrations of the hollow SiO_2_ nanoparticles. (H) Infrared images of stable state of the sandwich structure samples putting on a heating table set at 60°C for directly presenting the thermal insulation property of the HSPU nanocomposite films. The insets show that the HSPU nanocomposite film (HS_1.5_PU_10_) is put between two pieces of normal glass act as the insulating layer in the sandwich structure samples. A piece of glass with the same thickness as the HSPU nanocomposite film is chosen as the reference. (I) Surface temperature variation curve of the sandwich structure samples after they are put on the heating table.

### 2.2 Multi‐Responsive Shape‐Memory Effect of Actuation Unit

To enable the MSS freely adjustable operational mode under complex weather conditions, the melamine foam/Ag/polyurethane composite film of multi‐responsive shape‐memory effect is designed as the actuation unit. The melamine foam is utilized as the skeleton to construct the actuation unit because it exhibits excellent elasticity and anti‐fatigue characteristic that its initial shape still remains after 1000 times of the bending deformation (Figure S6A and Movie S1). Moreover, its storage modulus is 2.1 MPa (∼48.8°C) that can offer the actuation unit moderate stiffness at relatively higher temperature (Figure S6B,C). Most important, it can easily form continuous three‐dimensional conductive network via coating Ag nanowires to provide the actuation unit electrical‐responsive shape‐memory effect attributed to the electro‐thermal effect of the Ag nanowires (Figure 3A) [54]. Laboratory‐synthesized thermosetting polyurethane (LTPU) is employed as the matrix because of its low melting point (T_m‐LTPU_ = 36°C) and exceptional shape‐memory effect (Figure 3B and Movie S2). The shape fixity ratio 98.2% and shape recovery ratio 98.8% well maintain after 300 cycles of shape programming process (Figure 3C). In order to realize electrical‐responsive shape‐memory behavior of the actuation unit under low driving voltage, the electro‐thermal effect of the melamine foam with different Ag coating cycles is evaluated. As presented in Figure 3D,E, increasing the Ag coating cycles can greatly enhance the conductivity of the melamine foam/Ag composite film, and achieving more pronounced electro‐thermal effect [55]. Here, the melamine foam/Ag composite film with 6 cycles of coating is utilized to fabricate the actuation unit of multi‐responsive shape‐memory effect for several reasons. Under the voltage of 5 V, its temperature reaches up to 53.1°C, which is 17.1°C higher than the T_m‐LTPU_ and fully enough to stimulate the shape‐memory effect of the LTPU (Figure 3D) [56]. The electro‐thermal effect of the melamine foam/Ag composite film is almost unchanged when the Ag coating cycling number is over 6 times originated from the minor change in the conductivity (Figure 3E). In addition, it exhibits rapid electrical‐responsive behavior (Figure 3F and Movie S3). Under the voltage of 5 V, its temperature quickly raises to 47.6°C within 15 s and finally stably stays at about 52.0°C. After releasing the external voltage, its temperature fast drops to 24.4°C, far below the T_m‐LTPU_ = 36°C, within 8 s.

**FIGURE 3:**
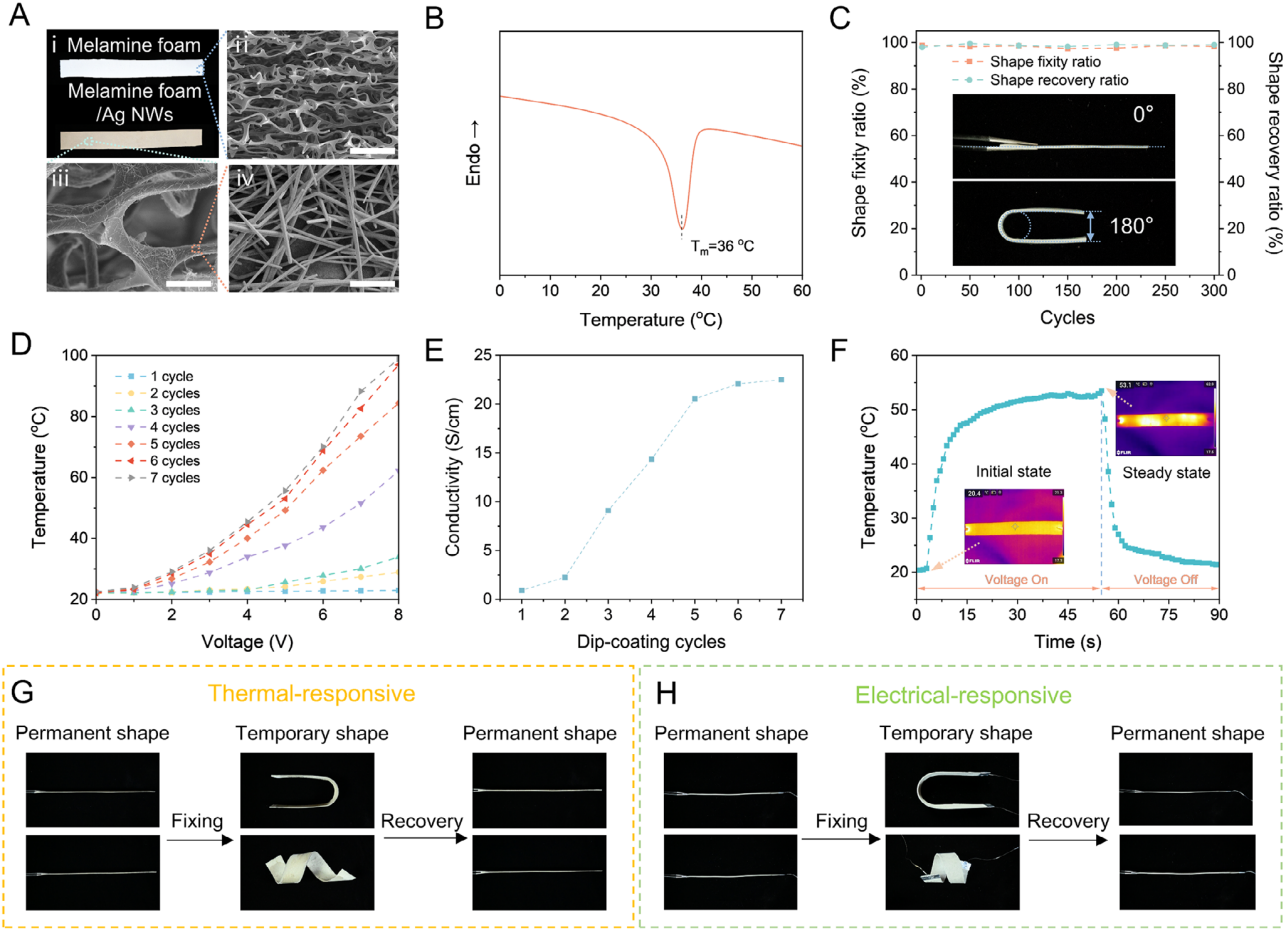
(A) i: Photographs of the melamine foam and melamine foam/Ag composite foam (6 cycles of Ag coating). ii and iii: Cross‐section SEM images the melamine foam and melamine foam/Ag composite foam (6 cycles of Ag coating). The scale bar of the (i) and (ii) is 100 µm and 10 µm, respectively. iv: SEM image of the Ag nanowires. The scale bar is 1 µm. (B) Differential Scanning Calorimetry (DSC) curve of the LTPU. (C) Stable shape‐memory effect of the LTPU. (D) Electro‐thermal effect and (E) conductivity of the melamine foam/Ag composite foams with different cycles of Ag coating. (F) Electrical‐responsive behavior of the melamine foam/Ag composite foam (6 cycles of Ag coating). (G) Shape‐memory effect and (H) electrical‐responsive shape‐memory effect of the actuation unit.

Figure 3G and Movie S4 demonstrate the shape‐memory effect of the actuation unit. During the programming process, flat actuation unit (permanent shape) is immersed into 50°C hot water to make its temperature over the T_m‐LTPU_. At high temperature (>T_m‐LTPU_), the flat actuation unit can be programmed to any temporary shape such as U or spiral shape under an external force due to its elasticity. When the actuation unit cools down to room temperature T_ambient_ = 26°C (<T_m‐LTPU_), the temporary shape of the actuation unit can be kept without the external force. The actuation unit is capable of recovering to the flat shape (permanent shape) as putting it into 50°C hot water again. As observed in Figure 3G and Movie S4, the change of ambient temperature is usually required to achieve the shape‐memory effect [57]. Thanks to the electrical‐responsive behavior of the actuation unit, the shape‐memory effect can be achieved under stable room temperature environment (<T_m‐LTPU_) assisted by the electrical field (Figure 3H and Movie S5).

### 2.3 Zero‐Energy Input Controllable Operation Mode of MSS in All‐Season

As presented in Figure 4A, a MSS assembled by three thermal managing units (15 cm × 15 cm) and two actuation units (15 cm × 1.8 cm) is employed as an example to demonstrate controllable operation mode in all‐season. For practical application, the MSS is linked to an aluminum alloy frame commonly used for the window encapsulation by two sliders. In hot season (T_ambient_>T_m‐LTPU_) (Figure 4Bi), the MSS is able to transform from expanded state to folded state through moving the sliders owing to the bending deformation of the elastic actuation units. The folded state can be facilely retained via locking the sliders to the aluminum alloy frame because the bended shape (temporary shape) of the actuation units can be held by the mechanical force. On the contrary, unlocking the sliders makes the MSS recover to expanded state. The operation principle for free switch of the MSS between the expanded state and folded state in hot season is based on the shape‐memory effect of the actuation units. This working principle also works for the MSS in cold season (T_ambient_<T_m‐LTPU_) attributed to electrical‐responsive shape‐memory effect of the actuation units (Figure 4Bii). Applying an external voltage (5 V), the temperature of the actuation units is able to raise over T_m‐LTPU_ for gaining the elasticity. The MSS can change from expanded state to folded state by moving the sliders to obtain the bended shape (temporary shape) of the actuation units. After removing the applied voltage, the folded state of the MSS maintains because the bended shape (temporary shape) of the actuation units can be memorized as their temperature falls to T_ambient_ (<T_m‐LTPU_). Applying the voltage again, high temperature (>T_m‐LTPU_) of the actuation units allows them return to the flat shape (permanent shape), leading the expanded state of the MSS. The MSS stably stays at the expanded state after releasing the applied voltage.

**FIGURE 4:**
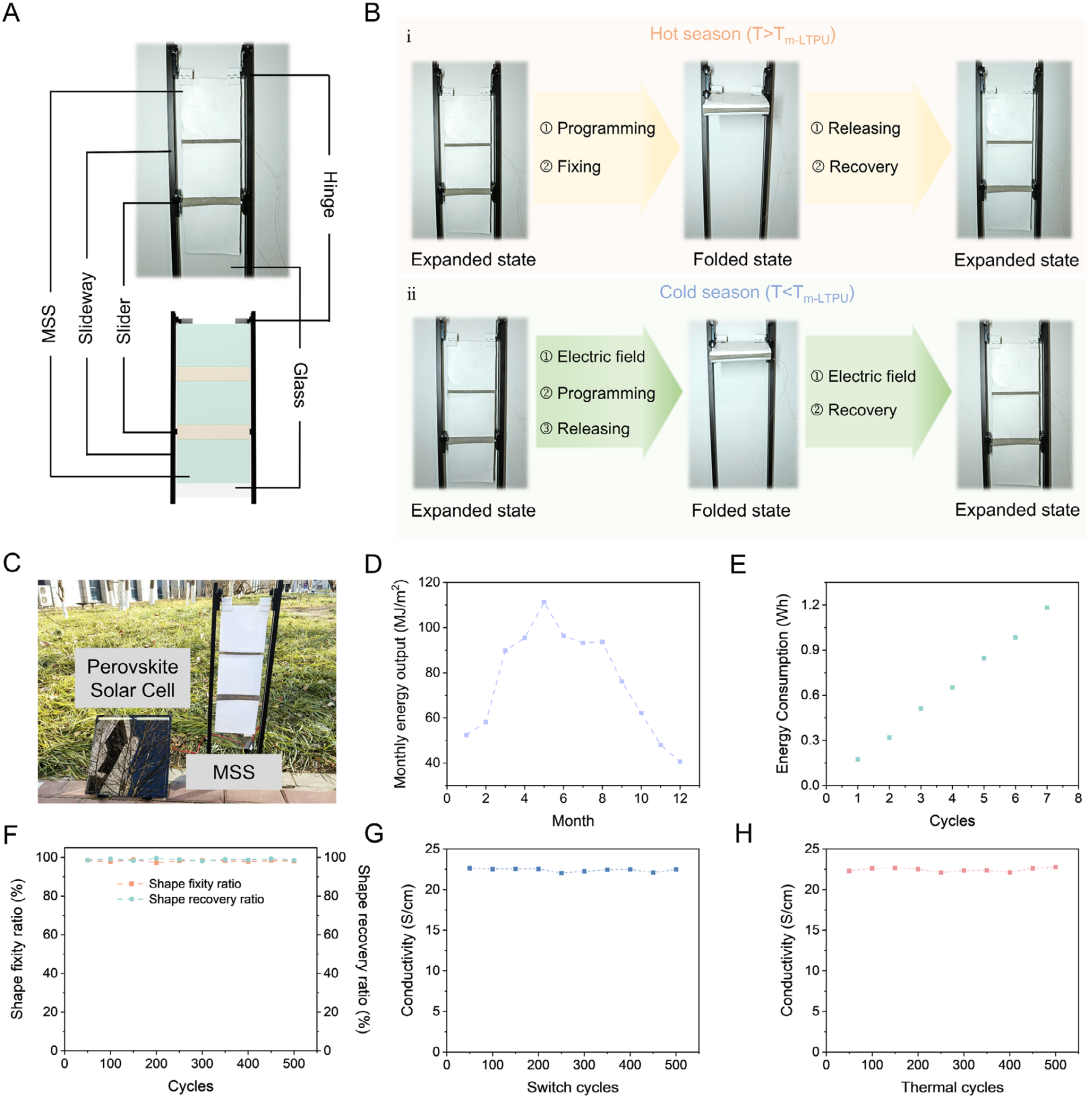
(A) Photograph and schematic of the setup for practical application of the MSS. (B) Controllable operation mode of the MSS in all‐season attributed to its multi‐responsive shape‐memory effect. (C) Photograph of the integration of the MSS with perovskite solar cell for enabling zero‐energy input controllable operation mode. (D) the monthly energy output of perovskite solar cell in Beijing (simulated by pvlib). (E) The electrical energy consumption by the MSS for 8 cycles via an electric field. (F) Stable shape‐memory effect of the actuation unit. The conductivity of the melamine foam/Ag composite foam (6 cycles of Ag coating) during (G) 500 times of the bending deformation and (H) thermal cycling.

Benefiting from multi‐responsive shape‐memory effect of the actuation units, the MSS can be freely and controllably switched between the expanded and folded states according to the demand of the occupant under different weather conditions. However, additional electric energy input is needed for the MSS to achieve all‐day controllable operation mode in cold season. To address this issue, a perovskite solar cell of stable photoelectrical performance is assembled with the MSS (Figure 4C and Figure S7). The perovskite solar cell can harness solar energy to generate the electric energy to afford the energy resource for stimulating electrical‐responsive shape‐memory effect of the actuation units in all‐day. According to the simulation (Figure 4D), annual power generation of the perovskite solar cell is able to power more than 1000 cycles shape programming process of the actuation unit (expanded state‐folded state‐expanded state) (Figure 4E). Therefore, all‐season zero‐energy input controllable operation mode of the MSS is achieved by the integration of the perovskite solar cell and MSS. Last but not the least, cycle and thermal stability tests concerning about the shape‐memory effect and conductivity of the MSS are performed to prove superior operational stability (Figure 4F–H).

### 2.4 Indoor and Outdoor Energy‐Saving Tests

To evaluate the energy‐saving performance of the MSS, a small “house” with a window is built up by utilizing an aluminum foil wrapped polystyrene foam box as the main body (Figure 5A). The chamber (8 cm × 8 cm × 8 cm) in the polystyrene foam box can be considered as the indoor environment. Its temperature is recorded by a K‐type thermocouple. In addition, xenon and infrared lamps are used as the solar simulator for the energy‐saving test [58]. As presented in Figure 5B, the chamber temperature sharply goes up from room temperature (T_ambient_ = 25.0°C) to T_fs_ = 50.5°C under the illumination of the xenon lamp as the MSS is at folded state. Under the same irradiation condition, the chamber temperature just increases to T_es_ = 33.9°C as the MSS is at expanded state. The large distinction (16.6°C) of the chamber temperature for the MSS at different states verifies extraordinary energy‐saving effect. In hot season, the MSS can greatly reduce the energy consumption for air‐conditioning by hindering the thermal irradiation of solar energy into the indoor because of its high solar reflectance (*R*_sol =_ 93.8%) at expanded state. In cold season, high *T*_sol =_ 88.2% of the MSS at folded state allows the thermal irradiation of solar energy to enter into the indoor for compensating the energy consumption for heating. The energy‐saving effect of the MSS closely related to its Δ*T*_sol_ is further validated by the energy‐saving test by applying the infrared lamp as the solar simulator (Figure 5C).

**FIGURE 5:**
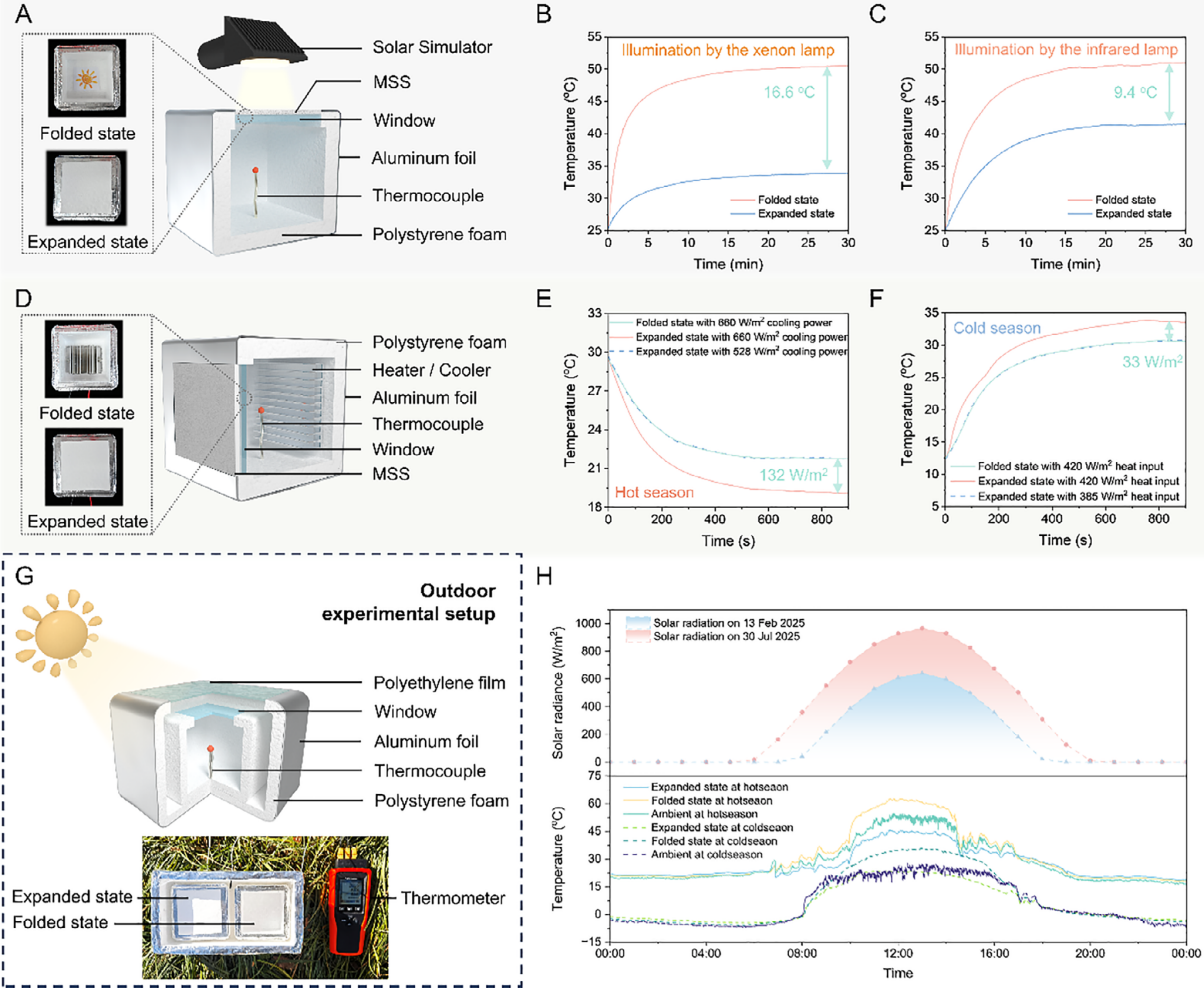
(A) Schematic of the setup for indoor tests to prove the energy‐saving effect determined by the Δ*T*_sol_ of the MSS. Temperature variation curves of the chamber during the process of the solar irradiation under different states of the MSS when utilizing (B) a xenon or (C) an infrared lamp as the solar simulator. (D) Schematic of the setup for indoor tests to demonstrate the energy‐saving effect dominated by the thermal insulation property of the MSS. Temperature variation curves of the chamber under the action of (E) the cooler and (F) heater for the MSS at different states. (G) Photograph and schematic of the setup for outdoor tests. (H) Upper part: real‐time solar irradiance of one day in hot season (30 July) and one day in cold season (13 February) at Beijing. Lower part: real‐time temperature of the chamber in these two days for the MSS at different states. The ambient real‐time temperature for these two days is recorded for reference.

In our design, the thermal insulation property of the MSS also takes an essential role in the energy‐saving performance. As presented in Figure 5D, a heater or a cooler is put into the chamber for adjusting its temperature. In hot season (Figure 5E), the chamber temperature can decrease from room temperature T_ambient_ = 29.7°C to 19.1°C under the cooling power of 660 W/m^2^ when the MSS is at expanded state. Under the same cooling power, the chamber temperature cools down to 21.8°C as the MSS is at folded state. Compared to the MSS at folded state, the MSS at expanded state displays more outstanding cooling effect because the thermal conductivity of the MSS (ĸ = 54 mW/m·K) is much smaller that of the glass (ĸ = 91 mW/m·K). The distinguished thermal insulation performance of the MSS is beneficial for saving the energy consumption for air‐conditioning in hot season which is demonstrated by the fact that the chamber temperature is able to low to 21.8°C under the cooling power of 528 W/m^2^ as the MSS is at expanded state. Here, the energy‐saving mechanism of the MSS at expanded state is effective inhibition the heat exchange between the indoor and outdoor environment. Therefore, the MSS at expanded state can also reduce the energy consumption for heating in cold season at the nighttime (Figure 5F).

According to the outdoor testing results in different seasons (Figure 5G,H), the operation principle for the MSS to afford exceptional energy‐saving performance is illustrated. During the daytime in all‐season, the temperature variation of the chamber for the MSS at different states gradually increases with the augment of the solar irradiation intensity. The maximum temperature variation (∼16.7°C) emerges as the solar irradiance displays the strongest intensity. As a result, the Δ*T*_sol_ of the MSS takes a dominating role in the energy‐saving performance. During the daytime in hot season, the MSS at expanded state is able to reflect the thermal irradiation of solar energy as to save the energy consumption for cooling. During the daytime in cold season, the MSS at folded state can compensate the energy consumption for heating by harnessing solar energy. On the other hand, the temperature variation of the chamber for the MSS at different states is no more than 1°C during the nighttime in all‐season (with no solar irradiation). Therefore, the thermal insulation property, instead of the Δ*T*_sol_, of the MSS plays the main role in the energy‐saving performance. During the nighttime in all‐season, the MSS at the expanded state is capable of effectively curb the heat exchange between the indoor and outdoor environment for greatly reducing the energy consumption for air‐conditioning and heating. It is worthwhile to point out that the energy‐saving performance of the MSS during the daytime in hot season can be further enhanced by the thermal insulation property.

### 2.5 Thermal Modelling and Energy‐Saving Evaluation

To further explore the energy‐saving potential and carbon dioxide reduction of the MSS in buildings, the simulation is performed based on the device properties and climate database by EnergyPlus (see Materials and Methods for details) [59]. In order to comprehensively illustrate the energy‐saving capabilities of the MSS, annual building energy consumption for air‐conditioning and heating of twenty‐two cities with distinct climatic conditions is estimated (Figure 6A and Figure S8). The normal glass is used as the reference. As presented in Figure 6B,C, the employment of the MSS can afford indoor thermal comfort meanwhile substantially reducing the seasonal energy demand, consequently making contribution to global energy saving and carbon emission mitigation. In these cities, Riyadh, Cario, Los Angles, and Sydney possess relatively higher energy‐saving efficiency (>30%) and carbon emission reduction (>60 kg/m^2^) because of their typical climate condition of high temperature and solar radiation [60]. In contrast, relatively lower energy‐saving efficiency (<20%) and carbon emission reduction (<35 kg/m^2^) are observed in the cities like Oslo, Ottawa, Berlin and Moscow which have inherently mild climate or long cold period. On a global average, the application of the MSS to the window is estimated to reduce carbon emissions by approximately 58.3 kg/m^2^, along with average energy‐saving efficiency of 26.7%. To demonstrate substantial potential of the MSS in lowering building energy consumption, the energy‐saving effect of the MSS is compared to Low‐E glass practically used in ESBs [61]. According to the simulating result, average energy‐saving efficiency and carbon emission reduction of commercial Low‐E glass are 7.6% and 0.4 kg/m^2^ verifying extraordinary energy‐saving performance of our designed MSS.

**FIGURE 6:**
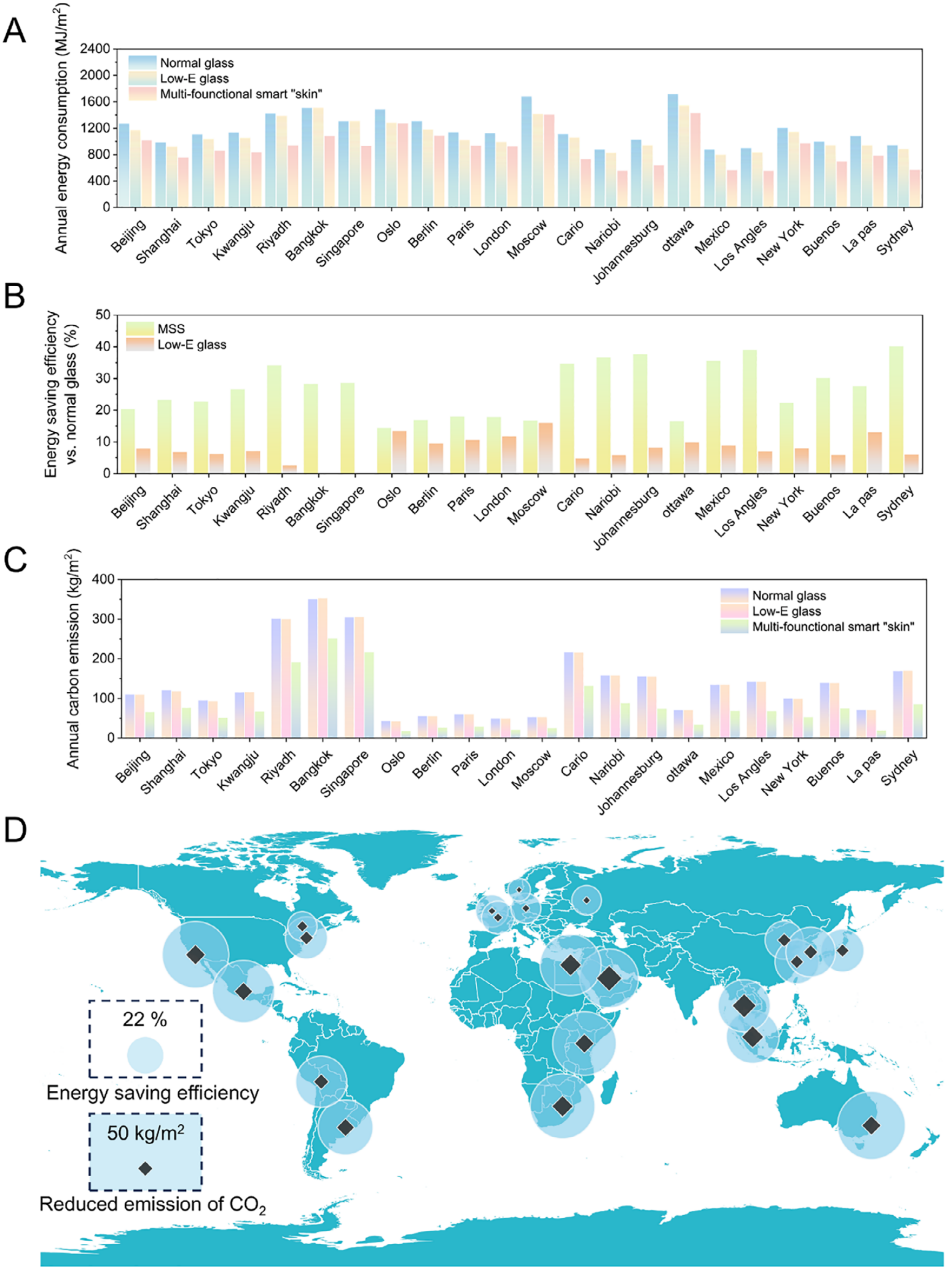
(A) Annual cooling and heating energy consumption of 22 cities with distinct climate conditions as applying the MSS, commercial Low‐E glass, and normal glass in buildings. (B) Annual energy‐saving efficiency of the MSS and commercial Low‐E glass relative to normal glass across twenty‐two global cities. (C) Annual carbon emission of twenty‐two cities with distinct climate conditions as applying the MSS, commercial Low‐E glass, and normal glass in buildings. (D) Global energy‐saving map of the building model with the MSS. (Energy‐saving efficiency is proportional to the diameter of blue circles, while carbon emission reduction correlates proportionally with the diagonal length of diamonds).

## 3 Conclusions

In summary, we have demonstrated a MSS exhibiting exceptional energy‐saving performance and controllable operation mode in all‐season benefiting from high solar reflectance and low thermal conductivity of the thermal managing unit and multi‐responsive shape‐memory effect of the actuation unit. According to the simulating results by EnergyPlus (window‐to‐wall ratio of 33%) for twenty‐two cities of different climate conditions, the average energy‐saving efficiency and carbon emission reduction of the MSS is 26.7% and 58.3 kg/m^2^. These values can be further enhanced because high‐rise buildings with glass curtain wall (window‐to‐wall ratio ∼60%) gradually become a trend considering natural lighting, structural optimization, and aesthetic [62]. The MSS holds a great potential for real‐world application due to low cost (8.45$/m^2^) (Tables S1 and S2), scalable manufacturing technology, controllable operation mode in all‐season, excellent Δ*T*_sol_ and cycling stability (Table S3). Looking forward, the achievement of broadband modulation covering both solar and mid‐infrared spectrum would further improve the thermal management performance. In addition, the exploration of green manufacture technology and eco‐friendly materials to exploit the MSS paves the way for more sustainable and energy‐efficient urban environment.

## 4 Experimental Section

### 4.1 Materials

TPU pellets (150 A) were purchased from Dongguan Zhangmutou Ruixiang Polymer Materials Trading Co., Ltd. Hollow SiO_2_ nanoparticles were obtained from Yumu (Ningbo) New Materials Co., Ltd. Silver nitrate and acetone were supplied by Sinopharm Chemical Reagent Co., Ltd. Poly(vinyl pyrrolidone) (PVP, M_w_ = 1300000) was purchased from Tianjin Xiens Oped Technology Co., Ltd. Sodium chloride and butyl acetate were obtained from Shanghai Titan Technology Co., Ltd. Melamine foam (ρ = 25 mg/cm^3^) was purchased from Shanghai Tuhong New Materials Co., Ltd. Polycaprolactone diol (PCL, M_w_ = 2000) was purchased from Shanghai Bide Pharmatech Co., Ltd. Hexamethylene diisocyanate was supplied by Shanghai Macklin Biochemical Co., Ltd. Poly(hexamethylene diisocyanate) was purchased from Shanghai Myril Biochemical Technology Co., Ltd. Stannous octoate was obtained from Anhui Zesheng Technology Co., Ltd. N, N‐dimethylformamide, ethylene glycol, and ethanol were purchased from Aladdin Reagent Co., Ltd. All chemicals were used as received without further purification unless otherwise specified.

### 4.2 Preparation of HSPU Nanocomposite Films

HSPU nanocomposite films were prepared by a typical non‐solvent‐induced phase separation method. First, TPU pellets were dissolved in N, N‐dimethylformamide, acting as the precursor solution. Then, a certain content of hollow SiO_2_ nanoparticles were added into the precursor solution to form a homogeneous mixed solution. The mixed solution was cast into a mold and subsequently immersed in a water bath to induce the phase separation. The solvent exchange process was maintained for 2 h. Finally, the HSPU nanocomposite films were obtained by drying in an oven at 40°C for 12 h to completely remove the solvent. The HSPU nanocomposite films were labelled as HS_x_PU_y_. The x and y are loading of the hollow SiO_2_ nanoparticles and concentration of the TPU in the precursor solution. The pure porous TPU films were prepared by the same procedure for fabricating the HSPU nanocomposite films just without the addition of the hollow SiO_2_ nanoparticles. Comprehensive consideration of the solar energy reflecting and thermal insulation property, the HS_1.5_PU_10_ was selected as the thermal managing unit to build up the MSS.

### 4.3 Preparation of Silver Nanowires (AgNWs)

The AgNWs were synthesized by hydrothermal method. Typically, 0.85 g poly(vinyl pyrrolidone) and 2.3 mg sodium chloride were dissolved in 20 mL of ethylene glycol and heated to 120°C. Then, 30 mL ethylene glycol solution containing 0.4 g silver nitrate was added dropwise into the above solution under continuous stirring. The precursor solution was obtained after 5 min. Afterwards, the precursor was transferred into a 100 mL polytetrafluoroethylene (PTFE)‐lined autoclave and maintained at 160°C for 7 h. Finally, the AgNWs were obtained via the centrifugation and washing by distilled water and ethanol.

### 4.4 Preparation of PU Films with Shape‐Memory Effect

Briefly, 2.0 g polycaprolactone diol was placed into a glass vial and heated to 100°C. Subsequently, 2 mL butyl acetate, poly(hexamethylene diisocyanate), hexamethylene diisocyanate, and 0.5 wt.% stannous octoate were added into the polycaprolactone diol and stirred for several minutes to form the prepolymer mixture. The prepolymer mixture was poured into an aluminum mold and cured at 60°C for 2 h. Finally, the PU films with shape‐memory effect were obtained by drying at 80°C overnight.

### 4.5 Preparation of Melamine Foam/Ag/Polyurethane Composite Films with Multi‐Responsive Shape‐Memory Effect

To enable the PU films multi‐responsive shape‐memory effect, continuous three‐dimensional conductive network was initially constructed by using melamine foam and AgNWs as the skeleton and conducting functional component. The continuous three‐dimensional conductive network (melamine foam/Ag composite film) was prepared by a dip‐coating process. Typically, the AgNWs were first dispersed into the ethanol to obtain a suspension with a concentration of 5 mg/mL. The melamine foam/Ag composite film was fabricated by immersing the melamine foam into the AgNWs suspension for 5 min and drying in an oven at 70°C for 10 min. This process is defined as one dip‐coating cycle. The melamine foam/Ag/polyurethane composite films with multi‐responsive shape‐memory effect were prepared by immersing melamine foam/Ag composite film into the PU prepolymer and curing at 60°C for 24 h. Herein, the melamine foam/Ag/polyurethane composite films with 6 cycle dip‐coating process are utilized as the actuation unite of multi‐responsive shape‐memory effect.

### 4.6 Building Energy Simulation

A model house with dimensions of 8 m × 8 m × 3 m (length × width × height) is established in EnergyPlus. Each wall contains a centrally located window measuring 4 m × 2 m, resulting in a window‐to‐wall ratio of 33%. Heating and cooling are provided by an “ideal load air system” serving the entire zone. The building's energy consumption for heating and cooling is simulated using a full‐building EnergyPlus model. Indoor temperatures are maintained at 22°C during heating and 24°C during cooling. An air infiltration rate of 0.3 air changes per hour (ACH) is applied, in accordance with ASHRAE 90.1 and ASHRAE 62.1 standards. For carbon emissions calculations, the emission factors are set as 0.84 kgCO_2_/kWh for electricity and 0.056 kgCO_2_/MJ for natural gas.

### 4.7 Characterization

The Vis‐NIR transmittance and reflectance spectra of all samples were measured using a UV‐Vis‐NIR spectrophotometer equipped with an integrating sphere (PerkinElmer, Lambda 1050+). The solar transmittance (*T*_sol_) was calculated according to: $T_{\mathrm{sol}}=\int_{0.3\;\mu m}^{2.5\;\mu m}T\left(\lambda \right)\psi_{\mathrm{sol}}\left(\lambda \right)d\lambda /\int_{0.3\;\mu m}^{2.5\;\mu m}\psi_{\mathrm{sol}}\left(\lambda \right)d\lambda$where T(*λ*) is the measured spectral transmittance and *ψ*_sol_(*λ*) is the solar spectral irradiance under AM 1.5G conditions.

The microstructure of the HSPU nanocomposite films, melamine foam, melamine foam/Ag composite films, hollow SiO_2_ nanoparticles, and Ag nanowires were characterized by field‐emission scanning electron microscopy (FE‐SEM, Carl Zeiss, SUPRA 55 SAPPHIRE). The microstructure of the hollow SiO_2_ nanoparticles was characterized by transmission electron microscope (TEM, JEOL, JEM‐1400). The thermal conductivity of the HSPU nanocomposite films was measured using a Hot Disk (TPS 2500S). An infrared camera (FLIR E54) was employed to evaluate the thermal insulation performance of HSPU and the electro‐thermal effect of the actuation unit. The melting point of the shape‐memory PU films was determined using differential scanning calorimetry (DSC, NETZSCH DSC 214) at heating/cooling rates of 10°C min^−1^. The electrical conductivity of the melamine foam/Ag composite films was measured using a resistance/capacitance measuring device (TruEbox 01 RC).

### 4.8 Shape‐Memory Effect

The shape‐memory performance was evaluated based on the bending test. The samples were first immersed into a water bath of 50°C and bent to 180° under an external force. Then, they were taken out of the water bath and cooled down to the room temperature under the external force to fix the temporary shape. Afterwards, the bending angle (*θ*_1_) of the samples was recorded after removing the external force. Finally, the samples were immersed into a water bath of 50°C again to complete shape recover process. The bending angle (*θ*_2_) of the samples after the shape recover process was measured.

The shape‐memory performance including the shape fixity ratio (*R*_f_) and shape recovery ratio (*R*_r_) was determined according to the following equations:

$$R_{f}=\frac{\theta_{1}}{180^{\circ }}\;\times 100\%$$

$$R_{r}=\frac{180^{\circ }-\theta_{2}}{180^{\circ }}\;\times 100\%$$

## Author Contributions

**Zongxu Na**: investigation, visualization. **Li Yu**: conceptualization, methodology, writing – original draft, writing – review and editing. **Bo Lu**: project administration, funding acquisition, writing – review and editing. **Jing Li**: resources, validation. **Xianyang Yue**: validation, resources. **Qian Wang**: conceptualization, methodology, writing – review and editing, project administration, funding acquisition.

## Conflicts of Interest

The authors declare no conflicts of interest.

## Supporting information




**Supporting File 1**: advs78166‐sup‐0001‐SuppMat.docx.

**Supporting File 2**: advs78166‐sup‐0002‐MovieS1.mp4.

**Supporting File 3**: advs78166‐sup‐0003‐MovieS2.mp4.

**Supporting File 3**: advs78166‐sup‐0004‐MovieS3.mp4.

**Supporting File 4**: advs78166‐sup‐0005‐MovieS4.mp4.

**Supporting File 5**: advs78166‐sup‐0006‐MovieS5.mp4.

## Data Availability

The data supporting the findings are available within the article and its Supplementary Information. All data are available from the corresponding author upon request. Source data are provided with this paper.
