# Supplementary material for: Multi‐Functional Smart Skin for High‐Efficiency Energy‐Saving Buildings
Source: Adv Sci (Weinh). 2026 Oct 6:e78166. Online ahead of print. doi: 10.1002/advs.78166 (PMC13643015; doi:10.1002/advs.78166)
Supplement: Supplementary file 1 [file ADVS-9999-e78166-s005.docx]

Supporting Information

Multi-functional Smart Skin for High-efficiency Energy-Saving Buildings

Zongxu Na^1,3^, Li Yu^1*^, Bo Lu^2*^, Jing Li^3^, Xianyang Yue^3^, Qian Wang^3*^

^1^State Key Laboratory of Green and Efficient Development of Phosphorus Resources, Hubei Key Laboratory of Plasma Chemistry and Advanced Materials, School of Materials Science and Engineering, Wuhan Institute of Technology, Wuhan 430205, China.

^2^School of Printing and Packaging Engineering, Beijing Institute of Graphic Communication Beijing 102600, PR China.

^3^Institute for Advanced Materials and Technology, University of Science and Technology Beijing, Beijing 100083, PR China.

Correspondence to: Li Yu ([li.yu@wit.edu.cn](mailto:li.yu@wit.edu.cn)), Bo Lu (lubo@bigc.edu.cn), Qian Wang (b2286713@ustb.edu.cn),

**This PDF file includes:**

Figure S1 to S8

Table S1 to S3

Legend for Movie S1 to S5

**Other Supplementary Materials for this manuscript include the following:**

Movie S1 to S5

**Figure S1.** **A** Reflectance spectra, **B** reflectance variation curve, **C** thermal conductivity and **D** porosity of the porous TPU films with different TPU precursor solutions.

**Figure S2. A-E** Cross-section SEM images of the porous TPU films with different TPU precursor solutions. **F** High-magnification SEM image of the selected region in **C**, showing the detailed microstructure of the porous TPU film (10 wt%).

**Figure S3.** **A-E** Cross-section SEM images and **F** porosity of the porous HSPU nanocomposite films with different concentrations of the hollow SiO_2_ nanoparticles.

**Figure S4.** **A** Infrared images of stable state of the sandwich structure samples putting on a heating table set at 60 ^o^C for directly presenting the thermal insulation property of the HSPU nanocomposite films with different concentrations of the hollow SiO_2_ nanoparticles. **B** Surface temperature variation curve of the sandwich structure samples from putting on the heating table to reaching the stable state. **C** Temperature difference of the surface temperature of the sandwich structure samples at stable state.

**Figure S5.** **A** Photograph of the HSPU nanocomposite film (HS_1.5_PU_10_) with the length and width of 20 cm respectively. **B** Solar reflectance and **C** thermal conductivity of three distinct areas in the prepared HSPU nanocomposite film.

**Figure S6.** **A** Elasticity and anti-fatigue characteristic of the melamine foam. **B** Dynamic thermomechanical analysis (DMA) curves of the melamine foam. **C** Mechanical stability of the actuation unit after the loading (20 g) at relatively higher temperature of 51.2 °C.

**Figure S7.** Current-voltage (I-V) curve of the perovskite solar cell under MSS switching stress.

**Figure S8.** Simulating monthly cooling and heating energy consumption of twenty-two cities with distinct climate conditions as applying the MSS and commercial Low-E in buildings. The normal glass is selected as the reference.

**Table S1.** The cost for the preparation of the MSS.

| Sample | Raw material price | Cost  /Square meter |
| --- | --- | --- |
| Thermoplastic polyurethane | $2459.52/t | $0.34 |
| N, N-dimethylformamide | $570.96/t | $0.72 |
| Hollow SiO₂ nanoparticles | $2.93/g | $6.15 |
| Melamine foam | $14.64/m^2^ | $1.21 |
| AgNWs | $600.24/kg | $0.003 |
| PU films with shape-memory effect | $8198.40/t | $0.029 |
| Total | | $8.45 |

**Table S2.** The cost of some commercial energy-saving glass compared to this work.

| Sample | Manufacturer | Cost  /Square meter |
| --- | --- | --- |
| **MSS** | - | **$8.45** |
| PDLC | Zhejiang Kangmingdun New Material Technology Co., Ltd. | $61.93 |
| PDLC | Wanruinaisi Technology Co., Ltd. | $33.82 |
| EC glass^[21,22]^ | Sage, View Smart Windows et.al | $538.00~1076.00 |
| Low-E glass^[23]^ | Shandong Jinjing Science & Technology Stock Co., Ltd. | $50.00 |
| Low-E glass^[23]^ | Beijing Bright View Windows and Glass Co., Ltd. | $150.00 |

**Table S3.** Comparison of optical properties, solar modulation performance, driving mode and cycling stability of the MSS with current smart devices.

| Reference | Base material | *T*_lum_ / % | Δ*T*_sol_ / % | Driving mode | Cycling stability |
| --- | --- | --- | --- | --- | --- |
| **This work** | **HSPU nanocomposite** | **90.2** | **88.1** | **Electrical & mechanical** | **500** |
| [1] | VO_2_ | 46.1 | 19.4 | Thermal | - |
| [2] | VO_2_ | 53.2 | 15.9 | Thermal | - |
| [3] | VO_2_ | 57.4 | 18.5 | Thermal | 7000 |
| [4] | VO_2_ | 75.4 | 11.5 | Thermal | - |
| [5] | VO_2_ | 76.8 | 21.0 | Thermal | - |
| [6] | Hydrogel | 87.2 | 81.3 | Thermal | 1000 |
| [7] | Hydrogel | 90.0 | 68.1 | Thermal | 100 |
| [8] | Hydrogel | 92.5 | 77.2 | Thermal | 100 |
| [9] | Hydrogel | 92.7 | 82.1 | Thermal | 100 |
| [10] | Hydrogel | 97.9 | 81.7 | Thermal | 1000 |
| [11] | Perovskite | 83.2 | 23.7 | Thermal | - |
| [12] | Perovskite | 85.5 | 23.0 | Thermal | 50 |
| [13] | Perovskite | 82.0 | 30.9 | Thermal | 10 |
| [14] | Perovskite | 66.8 | 33.3 | Thermal | 20 |
| [15] | Perovskite | 83.7 | 24.7 | Thermal | 240 |
| [16] | PD&SLCs | 55.3 | 40.9 | Thermal & Electrical | - |
| [17] | PD&SLCs | 57.8 | 34.6 | Thermal & Electrical | 30 |
| [18] | PDLCs | 79.7 | 25.9 | Thermal & Electrical | 150 |
| [19] | PDLCs | 48.5 | 56.1 | Electrical | 100 |
| [20] | PDLCs | 45.3 | 51.9 | Electrical | 200 |

**Legend for Movies**

**Movie S1.** Elasticity and anti-fatigue characteristics of the melamine foam.

**Movie S2.** Shape-memory effect of the laboratory-synthesized thermosetting polyurethane (LTSPU).

**Movie S3.** Electrical-responsive behavior of the melamine foam/Ag composite foam with 6 cycles of Ag coating.

**Movie S4.** Shape-memory effect of the actuation unit.

**Movie S5.** Electrical-responsive shape-memory effect of the actuation unit.

**Supplementary References**

1. L. Wu, A. Teng, M. Li, et al., “Kirkendall effect induced ultrafine VOOH nanoparticles and their transformation into VO_2_(M) for energy-efficient smart windows,” *Materials Horizons* 11, no. 4 (2024): 1098-1107. <https://doi.org/10.1039/D3MH01393F>
2. J. Pi, C.-B. Li, R.-Y. Sun, et al., “Superhydrophobic and thermochromic VO_2_-Based composite coatings for energy-saving smart windows,” *Composites Communications* 32, (2022): 101167. <https://doi.org/10.1016/j.coco.2022.101167>
3. Z. Chen, Y. Tang, A. Ji, et al., “Large-Scale Preparation of Durable VO_2_ Nanocomposite Coatings,” *ACS Applied Nano Materials* 4, no. 4 (2021): 4048-4054. <https://doi.org/10.1021/acsanm.1c00387>
4. Y. Su, J. He, “A confined VO_2_ synthesis plus liquid-like shielding strategy for durable, omniphobic, and multifunctional smart windows,” *Chemical Engineering Journal* 519, (2025): 165146. <https://doi.org/10.1016/j.cej.2025.165146>
5. S. Zhao, Z. Shao, A. Huang, et al., “Dynamic full-color tunability of high-performance smart windows utilizing absorption-emission effect,” *Nano Energy* 89, (2021): 106297. <https://doi.org/10.1016/j.nanoen.2021.106297>
6. X.-H. Li, C. Liu, S.-P. Feng, et al., “Broadband Light Management with Thermochromic Hydrogel Microparticles for Smart Windows,” *Joule* 3, no. 1 (2019): 290-302. <https://doi.org/10.1016/j.joule.2018.10.019>
7. Y. Zhou, S. Wang, J. Peng, et al., “Liquid Thermo-Responsive Smart Window Derived from Hydrogel,” *Joule* 4, no. 11 (2020): 2458-2474. <https://doi.org/10.1016/j.joule.2020.09.001>
8. R. Zhang, B. Xiang, Y. Shen, et al., “Energy-efficient smart window based on a thermochromic microgel with ultrahigh visible transparency and infrared transmittance modulation,” *Journal of Materials Chemistry A* 9, no. 32 (2021): 17481-17491. <https://doi.org/10.1039/D1TA03917B>
9. W. Gao, L. Wang, Q. Wei, et al., “Pure Physical-Crosslinked High-Strength Thermochromic Hydrogel for Smart Window and Energy Conservation,” *Advanced Functional Materials* 35, no. 16 (2025): 2418941. <https://doi.org/10.1002/adfm.202418941>
10. B. Yang, E. Zhou, W. Lv, et al., “Thermochromic hydrogels for synergistic mechano-optical properties and global energy saving potential,” *Nature Communications* 16, no. 1 (2025): 10080. <https://doi.org/10.1038/s41467-025-65071-w>
11. S. Liu, Y. Li, Y. Wang, et al., “Mask-inspired moisture-transmitting and durable thermochromic perovskite smart windows,” *Nature Communications* 15, no. 1 (2024): 876. <https://doi.org/10.1038/s41467-024-45047-y>
12. S. Liu, Y. W. Du, C. Y. Tso, et al., “Organic Hybrid Perovskite (MAPbI_3−x_Cl_x_) for Thermochromic Smart Window with Strong Optical Regulation Ability, Low Transition Temperature, and Narrow Hysteresis Width,” *Advanced Functional Materials* 31, no. 26 (2021): 2010426. <https://doi.org/10.1002/adfm.202010426>
13. J. Lin, M. Lai, L. Dou, et al., “Thermochromic halide perovskite solar cells,” *Nature Materials* 17, no. 3 (2018): 261-267. <https://doi.org/10.1038/s41563-017-0006-0>
14. L. M. Wheeler, D. T. Moore, R. Ihly, et al., “Switchable photovoltaic windows enabled by reversible photothermal complex dissociation from methylammonium lead iodide,” *Nature Communications* 8, no. 1 (2017): 1722. <https://doi.org/10.1038/s41467-017-01842-4>
15. H. He, S. Liu, Y. Du, et al., “Enhancing Stability: Two-Dimensional Thermochromic Perovskite for Smart Windows in Building Applications,” *Advanced Functional Materials* 36, no. 6 (2026): 2417582. <https://doi.org/10.1002/adfm.202417582>
16. X. Liang, M. Chen, Q. Wang, et al., “Active and passive modulation of solar light transmittance in a hybrid thermochromic soft-matter system for energy-saving smart window applications,” *Journal of Materials Chemistry C* 6, no. 26 (2018): 7054-7062. <https://doi.org/10.1039/C8TC01274A>
17. X. Liang, M. Chen, S. Guo, et al., “Dual-Band Modulation of Visible and Near-Infrared Light Transmittance in an All-Solution-Processed Hybrid Micro-Nano Composite Film,” *ACS Applied Materials & Interfaces* 9, no. 46 (2017): 40810-40819. <https://doi.org/10.1021/acsami.7b11582>
18. Z. Zhang, M. Yu, C. Ma, et al., “A Janus Smart Window for Temperature-Adaptive Radiative Cooling and Adjustable Solar Transmittance,” *Nano-Micro Letters* 17, no. 1 (2025): 233. <https://doi.org/10.1007/s40820-025-01740-1>
19. Y. Deng, Y. Yang, Y. Xiao, et al., “Ultrafast Switchable Passive Radiative Cooling Smart Windows with Synergistic Optical Modulation,” *Advanced Functional Materials* 33, no. 35 (2023): 2301319. <https://doi.org/10.1002/adfm.202301319>
20. Y. Deng, Y. Yang, Y. Xiao, et al., “Annual Energy-Saving Smart Windows with Actively Controllable Passive Radiative Cooling and Multimode Heating Regulation,” *Advanced Materials* 36, no. 27 (2024): 2401869. <https://doi.org/10.1002/adma.202401869>
21. PatSnap Eureka, “Evaluating Cost-Benefit of Electrochromic Glass in Modern Infrastructure,” Available at: [https://eureka.patsnap.com/report-evaluating-cost-benefit-of-electrochromic-glass-in-modern-infrastructure](https://eureka.patsnap.com/report-evaluating-cost-benefit-of-electrochromic-glass-in-modern-infrastructure?utm_source=chatgpt.com) (2026).
22. Colfax Glass, “Smart Windows & Electrochromic Glass: California Homeowner Guide,” Available at: <https://www.colfaxglass.com/blog/smart-windows-electrochromic-glass-california-2026?utm_source=chatgpt.com> (2026).
23. Z. Zhou, R. Liu, Z. Huang, et al., “Radiative Warming Glass for High-Latitude Cold Regions,” *Advanced Science* 12, no. 9 (2025): 2414192. <https://doi.org/10.1002/advs.202414192>
